# Knotting of Ileum by Meckel’s Diverticulum Leading to Acute Small Bowel Obstruction: An Exceptional Case

**DOI:** 10.4103/1319-3767.80392

**Published:** 2011

**Authors:** Vipul D. Yagnik, Bhargav D. Yagnik

**Affiliations:** Ronak Endo-Laparoscopy and General Surgical Hospital, Department of Surgical Gastroenterology, Patan, Gujarat, India; 1 General practitioner, Ahmedabad, Gujarat, India. E-mail: vipul.yagnik@gmail.com

**Figure 1:**
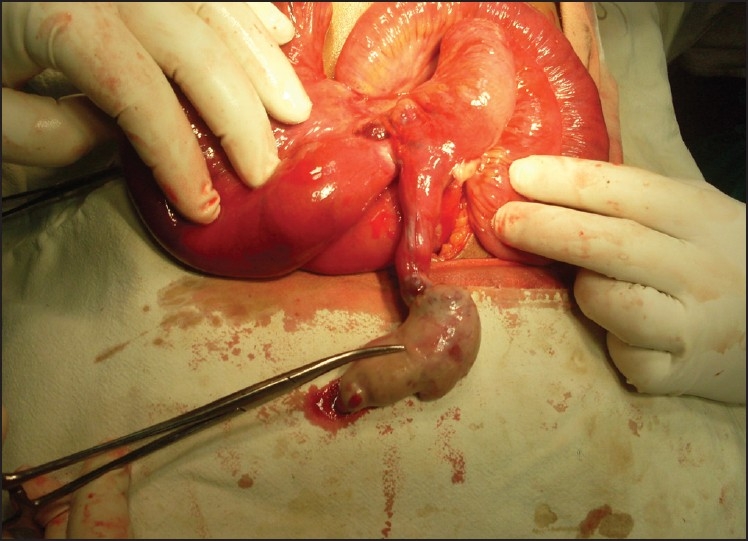
Released Meckel’s with constriction mark at the base withdilated proximal bowel

Sir,

A 12-year-old male came to the emergency room with complaints of right iliac fossa pain and abdominal distention since the last 48 h. This was associated with nausea, vomiting, and absolute constipation. Abdominal examination finding revealed a generalized distention with localized guarding and rigidity in the right lower quadrant. Bowel sounds were hyperperistaltic. Examination revealed BP of 100/70 mmHg, pulse rate of 100/min, and temperature of 38°C. Laboratory data showed white cell count of 20,000/mm ^3^with left shift. A plain radiograph of the abdomen erect revealed intestinal obstruction. On opening the abdomen, knotting of Meckel’s around the small bowel was seen. Knot was untied and the diverticulum released [Figure 1]. Resection of the gangrenous Meckel’s diverticulum and short segment of ileum and anastomosis was performed. Postoperative course was unremarkable.

Meckel’s diverticulum is the most frequent congenital anomaly of the gastrointestinal tract, is located on the antimesenteric border of ileum 2 ft proximal to the ileocecal junction. It is named after Johann Meckel, who described it in detail in 1809.[1] Meckel’s diverticulum is a true diverticulum, possesses all three coats of the intestinal wall, and has its own blood supply.[2] The size is variable with average length being 5 cm and the arterial supply is derived from the persistent vitelline artery arising from the superior mesenteric artery or less frequently ileocolic artery.[3] The majority of cases are asymptomatic. If symptomatic, it can present as bleeding per rectum, intestinal obstruction, or diverticulitis.

Knotting of ileum by Meckel’s diverticulum is a rare presentation of Meckel’s leading to acute small bowel obstruction.[4] This case is exceptionally unusual as knotting was not associated with internal hernia and only tip of the Meckel’s was gangrenous. After initial resuscitation, management of such cases includes either laparoscopic or open surgery.
